# Aging in Indigenous Communities: Perspective from Two Ancestral Communities in the Colombian Andean–Amazon Region

**DOI:** 10.1007/s10823-023-09495-1

**Published:** 2024-05-08

**Authors:** Hernán D. García, Wilson A. García, Carmen L. Curcio

**Affiliations:** 1 Hospital Pablo Tobón Uribe, St 78B #69-240, Medellín, Colombia; 2 Universidad del Quindío, Armenia, Colombia; 3 Universidad de Caldas, Manizales, Colombia

**Keywords:** Ethnogerontology, Indigenous, Symbolic Interactionism, Meaning of Aging

## Abstract

The phenomenon of world aging is not foreign to indigenous communities. In the last few years, research about these communities around the world has increased, but aging in indigenous towns still has not been studied widely. The purpose of this research is to interpret the meaning of old age in two indigenous communities from the Colombian Andean–Amazon region (the Inga and Kamëntsa) to reinforce the relevance of the local sociocultural context within the configuration of the meaning of old age and to emphasize the importance of considering particular regional characteristics for the design of policies and interventions aiming to recognize and integrate indigenous populations. This is a qualitative study with an interactionism–symbolism approach. In total, six indigenous people older than 60 years from two ancestral communities from the Colombian Andean–Amazon region participated in the in-depth interviews. Data analysis was carried out in three moments: discovery, coding, and relativization of the information. The results show that old age means wisdom, “I am wise,” which is supported in the cosmology and the trajectory of life, reinforces the identity and autonomy, and allows them to be agents in the dynamics of their communities from the “I do,” in other words, their roles as builders of the family–society and as guards of ancestral knowledge. The loss of this knowledge and the elements that it is composed of uproot them and put them at risk of disappearing as individuals and as a collective. In conclusion, the meaning of old age in these communities is not centered on a determinate age; you are not old, you are wise, and as such, they play a central role in their communities. Moreover, wisdom is built in parallel with their cosmology and assigns them the task of safekeeping ancestral knowledge. In order to do this, they use oral tradition as a tool, words that are born in their territories, travel in a nonlinear timeline, and get strengthened by the community while also protecting it and building it. Knowing what aging means for Indigenous communities can facilitate to the development of policies and initiatives and to provide culturally appropriate and effective programs.

## Introduction

It has been widely documented that the world is experiencing an unprecedented demographic transformation. Nowadays, demographic aging is an ongoing process, with variations among the regions of the world and among countries, but well-established nonetheless. From the viewpoint of (Quigley et al., 2022) its implications in all fields, it is perhaps the most important transformation of this era (CEPAL, 2018).

The indigenous population in Latin America is very diverse and represents more than one thousand communities with their own languages and dialects. Indigenous peoples have many distinctive characteristics that need special attention to understanding aging: different processes of populational aging, interwoven structural problems (including marked inequalities compared to the non-indigenous population), conflicts over land and territories, and cultural aspects related to how old age, aging, health and healthy living are envisaged and interpreted (Pan American Health Organization (PAHO, 2023). In most countries, indigenous peoples continue to live mostly in rural areas associated with their traditional territories, but there is marked heterogeneity between countries and between indigenous groups (Quigley et al., 2022). Indigenous peoples suffer the greatest structural inequalities in Latin America as a result of complex social, economic, cultural, and political situations that began during the Conquest and colonial period (PAHO, 2023).

One of the most frequently cited characterizations of indigenous status is included in the International Labour Organization’s Convention 169 (Organization, 1989), which incorporates traditional life styles, cultural expressions (including language and livelihoods), cultural and spiritual values associated with the land, distinct forms of social organization and political institutions, residence in clearly demarcated territories, and histories that predate colonization. Indigenous peoples hold a different worldview where the concept of self is collectivist and inseparable from land, family, and community (PAHO, 2023). Prior to meaningful interaction with European peoples in the sixteenth century, the ancient and protohistoric peoples of the Americas developed coherent, archaic worldviews. These worldviews typically have a number of tiered, axially arranged worlds that make up the cosmos. The fundamental elements of the environment, such as the sky, the ground, the subterranean realm, the waters, atmospheric processes, plants, animals, and more, are portrayed in these cosmologies as an integrated system that is fueled and controlled by the power of spirits and gods. These cosmologies are based on observation and interaction with nature. A way of thinking that organizes the social structure, political systems, economic systems, and religious expressions of the community is supported by the structure and order ascribed to the cosmos (Reichel, 1999).

The other hand, despite recent legislation protecting the human rights of indigenous people, their socioeconomic situation and wellbeing continues to be deficient compared to non-indigenous people. In addition, they are discriminated against, marginalized, and excluded from mainstream society. Due to their profound heterogeneity, indigenous aging varies enormously. They inhabit diverse communities and areas of residence, with distinct immigration statuses, levels of education, and so on (Cruz-Saco, 2018). Which are significantly different from those of non-indigenous populations (PAHO, 2023).

## Theoretical Framework

Indigenous peoples have metaphysical, unified, and egalitarian worldviews that are holistic, relational, emergent, and adaptable and include interdependent ecological/environmental, physical, emotional, and spiritual elements throughout all histories and civilizations.

Symbolic interactionism (SI) is a framework for developing theory that sees society as the product of everyday human interaction (Blumer, 1986). In other words, it provides a framework for understanding how people interact with one another to form symbolic worlds, and how these worlds in turn influence people’s behaviors. People create shared meanings through their interaction, and these meanings become their reality (Breton, 2012).

The following three fundamental principles offered by Blumer (Blumer, 1986) are taken into consideration in this study: (1) Humans respond in response to things in accordance with the meanings they ascribe to them; these meanings can vary depending on whether or not a person is an adult. (2) This presumption warns that the meaning does not derive from the essence of the things themselves, it is not an arbitrary construction of the individual subject, but is, above all, a social construction. The meaning of those things is derived from or arises as a result of the social interaction that each person maintains with their neighbor. (3) The meanings are manipulated through an interpretive process developed by the person when facing the things that he finds in his path; in other words, that every social actor inexorably transforms the meanings that he receives from the symbolic context that he inhabits. Similarly, Mead (1934) describes how the individual self is formed through the process of symbolic interaction with other individuals, in such a way that this interaction builds an individual identity. Symbolic interactionism plays a fundamental role in this process, since they allow individuals to interpret and assign meaning to objects and situations in their environment.

The Indigenous relationality principle is complemented by SI`s emphasis on interpersonal interactions as the cornerstone of society and the continuation of these interactions for the sustainability of a society. Indigenous communities, which are collectivistic cultures, depend on strong social bonds and interactions because they adopt a “I/We” mentality as opposed to a “I/You” mentality found in non-Indigenous, culturally individualistic societies (Chilisa, 2012). The “I/We” philosophy, which promotes “I am we,” “I am because we are,” and “a person is because of others’ relationships,” supports the “I/We” thinking in the Indigenous world. This philosophy is based on Indigenous concepts of commonality, unity, collectivity, plurality, and social justice (Chilisa, 2012). To put it another way, the idea that humans are the results of how they interact with their environment, SI is similar to indigenous cosmology: Indigenous worldviews are socially constructed.

### Aging in Indigenous Populations

There are various definitions of old age and aging, and these have various connotations across cultural boundaries. To explain these disparities, (Huenchuan, 2006) summarizes what the aging process means from an indigenous perspective, she distinguishes between three factors: one’s role, one’s social position, and one’s perception of aging. When it comes to defining the onset of old age, the author notes that, in contrast to non-indigenous cultures, where the chronological age is typically set at 60 years, for indigenous peoples physiological age predominates. This is because they have lost some instrumental and functional capacities needed to maintain their independence and autonomy. According to their stage of life and gender, each individual is given a position within the family in indigenous communities.

Older individuals have a responsibility to transmit their knowledge and culture to younger generations in these family- and culture-centered communities (Huenchuan, 2006). These persons are respected for their expertise and wisdom and are moral authorities and spiritual mentors. Older people play a participating and consultative role in decision-making that affects the entire society in indigenous communities, in contrast to non-indigenous civilizations where they frequently play a passive and dependent position. They are the ones who ultimately decide. Regarding one’s social standing as they become older. In families, men and women often rise in status with age, and reaching old age is frequently the highest social position a person—at least a man—can achieve (Huenchuan, 2006).

Older indigenous women typically only retain their status if they have worked as healers or midwives or if they share a civil or religious standing with their husbands (Ministerio de Cultura República de Colombia, 2010). The other women, who have experienced a lifetime of unfair opportunities, are neglected as they get older. This is especially true in urban areas, where conventional roles are vanishing and there is less regard for the elderly (Movimiento Regional Por la Tierra, 2015). Older people in indigenous communities participate more actively in the processes of production, working longer than non-indigenous people do (CEPAL, 2018).

### Context of the Study

Colombia is well-known for its cultural diversity even though its 115 indigenous groups represent only 4.4% of the country’s total population. The majority (79%) inhabit rural areas and there are no differences by sex (49.9% men and 50.1% women). 5.8% of the indigenous population overall is 65 or older. (Departamento Administrativo Nacional de Estadística - DANE, 2019). In Colombia the increased feminization of old age among indigenous peoples is less pronounced than in other Countries.

As it happens in other countries, older indigenous adults in Colombia share many of the vulnerabilities of their non-indigenous counterparts but in addition, they bear an additional burden imposed by ethnic status.(Cruz-Saco, 2018; DANE, 2019; PAHO, 2023). Indigenous people who are aging have the same unavoidable physical and mental decline as older members of other population groups, but the impacts of these processes are accelerated by socioeconomic factors associated to ethnic status, such as poverty, low levels of education, and other socioeconomic factors. These elements are linked to poor health outcomes and living in rural areas that are relatively isolated, where older indigenous adults must travel great distances and encounter other obstacles (such as inadequate roads and transportation networks) in order to easily access public services, markets, and healthcare (Kumar & Acanfora, 2001).

Both communities, Inga and Kamëntsa, live in the same area In the Colombian Andean–Amazon region, in a Sibundoy valley, in a tolerant and non-excluding manner, making it common to belong to both groups. They are widely recognized for their artisanal skill, shamanic knowledge, defense and development of their territory, and a strong organizational process, which have allowed them to have a very important social and political incidence in the region (Ministerio de Cultura República de Colombia, 2010).

In 2018, there were 19,561 people who self-identified as belonging to the Inga community and 6,5% older than 65 years (DANE, 2018). The inga community speak a dialect of Quechua known as Inga Kichwa. Spiritual guidance, belief, and traditional indigenous knowledge are fundamental to the culture of this culture. The Inga are descendants of an Inca colony that immigrated from the Peruvian tropical rainforest to Colombia. According to “The safekeeping plan of the Inga people: So that our lives and thought endure” (Ministerio del Interior, 2012) for the indigenous people, their territory is a source of life and inspiration. Mother Earth, Pacha Mama “NUKANCHIPA ALPA MAMA,” configures thought and actions, making the events that occur to Mother Earth a cause for collective worry.

Kamëntsa means “people from right here with their own thought and language” (Movimiento regional Por La Tierra, 2015), and previous data reported a population of around 4,879 indigenous people (Ministerio de Cultura, 2017). The survival of their memory and fight greatly depends on the preservation of what they consider their territory since ancient times and on a plan to safeguard its uses and customs, which is called “BËNGBE LUARENTŠ ŠBOACHANAK MOCHTABOASHËNTS JUABN, NEMORIA Y BËYAN,” which means “let’s plant our thought, memory, and language with strength and hope in our territory.” Its objective is to recover their own knowledge and wisdom, which have declined with time (Movimiento Regional Por la Tierra, 2015). The Sibundoy Valley’s social and cultural life is greatly influenced by the Kamsá craftsmen and their skill. Their social structure was centered on extended families and is now based on nuclear families. They have their own type of government called the council, however the “taitas” and “mamitas”, who are the oldest members of the community, hold the most legitimate authority.

Despite the symbolic and practical importance of elders in many indigenous cultures, older people in indigenous societies play a participatory and consultative role in problem-solving and decisions that affect the entire group (PAHO, 2023). However, there is a dearth of qualitative research on this group at the moment. Research on indigenous communities has increased in recent years in an effort to shed light on their diversity, customs, way of life, and generational structure, as well as their true needs and the function of elders in these societies. For these communities, life makes sense in their territories, where for hundreds or thousands of years they have built the symbolic references that give life meaning (Aristizábal Giraldo, 2015; Molinet Huechura, 2010). There, being born, growing up, reproducing, getting sick, aging, and dying acquire specific meanings within the context of each group. there is a relatively limited amount of research worldwide that has focused on health-related concerns of older Indigenous Peoples, including health and care access inequities (Goins et al., 2022). In this vein, the survey of health, nutrition, and wellbeing (SABE) Ecuador study evidenced there is a growing perception of old age as a state where opportunities, qualities, and hope are lost and that aimed at clarifying and understanding the perceptions, opinions, and concepts surrounding aging in an important part of the elder indigenous population in that country (Waters & Gallegos, 2012). The results show that for them identity is built through appreciation of the ability to perform physical activities. This aspect of identity goes against the inevitable deterioration and, as such, can affect their mental and physical health.

There is a growing recognition of the importance of subjective meaning of being elderly from a public policy perspective, and for their social and cultural relevance. However, the voices of elderly indigenous remain largely underrepresented in the emerging body of qualitative literature on aging. Given this gap, and Colombian’s burgeoning older population, this question constitutes a duty in a country where there is multiculturality and where there are legal promotion instruments for indigenous rights. Thus, the purpose of this research is to interpret the meaning of old age in two indigenous communities from the Colombian Andean–Amazon region (the Inga and Kamëntsa) to reinforce the relevance of the local sociocultural context within the configuration of the meaning of old age and to emphasize the importance of considering particular regional characteristics for the design of policies and interventions aiming to recognize and integrate indigenous populations.

## Methods

The IS was used as a perspective, which together with the qualitative methodology seeks to understand and interpret the social reality that is studied.

Participants. The study was carried out in Valle de Sibundoy in the Department of Putumayo (Colombia). We used opportunistic sampling and included the participation of six indigenous elders, three from the Kamëntsa community and three from the Inga community. There were four men and two women whose ages ranged between 60 and 77 years. With the premise that the interaction occurs within a particular social and cultural context in which social objects (persons), as well as situations, must be defined or categorized based on individual meanings (Blumer, 1986) and based on prior knowledge of the caste structure of the communities Inga y Kamëntsa, representatives of each of them were included: a political leader, a traditional doctor, an artisan, a musician, and a woman and a man from the community.

The inclusion criteria were that the participants be self-identified indigenous persons, being older than 60 years, consenting to participation through an informed consent, not being institutionalized, not having cognitive deterioration, not suffering from acute illness, and not having a communication, hearing, or speech disorder. They speak the kichwa and in most cases, Spanish as well.

Research Process and Ethics: Prior to the development of the research, we met with the authorities in the communities to present its objectives and to request permission to work with the town councilors and in their territory. An indigenous artisan leader served as “doorman,” facilitating the interaction with the participants. We obtained the approval of the community leaders and informed consent from each of the participants. After the study was approved by the bioethics committee of the Faculty of Health Sciences of the Universidad de Caldas, CBCS-062 of 2019.

Data collection. We selected individual in-depth interviews as the most pertinent technique, understood as a “digging tool.” Its main characteristic is generating accounts with which an approach to the opinions, knowledge, experiences, values, and perceptions about the world is achieved (Bonilla-Castro, 2005).

The semi-structured in-depth interviews were conducted at the interviewee’s home by one of the main researchers, always the same one, who also analyzed the information later. The interviews were conducted with open questions focused on the meaning of aging within the indigenous community. The interviews were carried out in a single session with no time limit and were carried out by a team of two people: a Spanish-speaking researcher and a bilingual indigenous person (Kichwa and Spanish), they were carried out in Spanish without the need for an interpreter/translator given the domain of the participants’ language. Although they all focused on the theme of aging, the questions were broad enough to allow participants to express the broadest possible range of views on the theme. The questions used during the interviews are as follows: In general, what does aging mean to you? What is the elders’ role in the community? What is your opinion of the elderly’s role in the community? What do you think about aging in your community?

Data Analysis. The data obtained using in-depth interviews, were recorded and transcribed, following Labrie’s recommendations (López Estrada & Deslauriers, 2011). Digitalized data were processed using ATLAS.ti version 8.3.17, making it possible to identify and code the categories in light of the theoretical and methodological approach following Taylor’s guidelines (Taylor and Bogdan, 1998).

Subsequently, we carried out an analytical-understanding phase from the interrelation of the identified descriptive categories and the construction of second-order or axial categories. Finally, we proceeded with relativization, where the triangulation by aggregates and interactive triangulation were done, given the presence of two different ethnics and the generation of the findings’ theses (collective triangulation). Wisdom, oral tradition, and connectedness to their territory are the three categories we now have recognized as explicative.

## Results

The results of this study are presented as a descriptive narrative and encompass the meaning of elders within the two studied indigenous communities, from the subjectivity of the participants, and their relation with their surroundings and society. According to the explicative categories three major themes were identified: (1) Wisdom: Elders are knowledge guardians, (2) Oral tradition: Safeguarding the knowledge of their ancestors through time and space. (3) Territoriality: Creating roots and uprooting.

### Wisdom: Elders are Knowledge Guardians

Elderly has a distinct significance for Inga and Kamëntsá people than it does in non-indigenous society. “In our culture we do not have that conception of old. There are the conditions of Taita (person of authority) or Tatsëmbuá (person of knowledge), Bacó or Batá (older man and woman), because we do not measure old age by the years” (A)^1^ Elders in Inga and Kamëntsa communities are considered guardians of ancestral knowledge, and they are in charge of its transmission to the community and future generations. The elders perceive themselves as wise beings or knower (sabedores) as a result of the information that has been preserved in memory and in that of their peoples.

Wisdom allows them to have a greater identity, not only as indigenous, but also as knowledgeable beings (sabedores), and through this construction, they strengthen their autonomy: doing, deciding, and freely choosing, looking for plenitude. All of this is framed within the concept of the life trajectory, from their birth, and even before, they are the product of their own, past, and collective experiences that reinforce that ancestral knowledge in various fields such as traditional medicine, craftsmanship, or agriculture: “Since I was little, I saw how my dad helped cure the sick” (TD); “my grandfather and my dad were artisans; they taught me to care for the earth” (CM).

For example, the traditional doctor is seen as wise and is in charge of healing the community. To do this, traditional doctors require many years of experience and practice, which is even rooted in their parents’, grandparents’, and the communities’ knowledge, and because of their profession, they are respected in the community: “The traditional doctor is celebrated by the community, very respected, loved, and cherished” (TD).

The belief in the tradition is that the traditional doctor has the expertise to safeguard humans, plants, animals, and other potential items from physical and mystical illnesses. In fact, they talk to him about the reasons why particular individuals or objects are affected. Knowledge is obtained empirically and by means of tradition; elders in Inga and Kamëntsa communities become the guardians of wisdom, and they are in charge of molding future generations. For indigenous people, knowledge comes from their Creator, and as such, it is not possible to deeply comprehend it if it is dealt with in isolation. For this reason, when someone with knowledge of oral tradition imparts that knowledge, he or she first declares the existence of the creator of his or her world and of his or her people before mentioning their ancestors, grandparents, aunts and uncles, and parents, from whom they inherited the knowledge from their own culture. “My grandma used to tell me her mom handled traditional plants, as did my grandfather; the knowledge passes from generation to generation” (TD).

The pillar of wisdom for Inga and Kamëntsa elders is the cosmology, which is the way to see and to see themselves in the world. One of the most relevant aspects is the experience within the community and the territory, but even more important within the cosmogonic space, where their roots, history memory, culture and traditions, mother tongue, losses and constructions, and reflections of their current dreams are. According to Inga and Kamëntsa cosmology, the territory is the place where you are born, you make a family, and you die; it is where you are closely related to what the land has to offer from the natural and supernatural planes; it is where wisdom has been transmitted for generations. “Pacha Mama has to be cared for. We should not destroy it or use chemical or herbicides” (P); “Respect for the land is so great that from a young age we are taught to use natural remedies to treat the crops” (CM).

From their view of wise beings, they also consider they are closer in the political development of their communities because their voice is heard and taken into account when making decisions that affect the community and during the election process of new leaders: “When I speak, I am heard,” “my voice is important” (P).

### Oral Tradition: Safeguarding the Knowledge of Their Ancestors Through Time and Space

In this study, we identified that, for the elders of these indigenous communities, old age is closely linked to a role change. When they become old, their role is to transmit the ancestral knowledge to the new generations, which involves various areas such as traditional medicine, handicraft, their mother tongue, and culture. “We must not lose the language because it is the tool to connect the community” (CM); “My dad taught us since we were young what plants were used for; what their powers were” (TD). For these communities, the concept of teaching is a flow of thought from one person to another for them to recognize the object or topic shown, and learning is to mentally see the objects or topics shown on their own.

We could evidence that elders in both indigenous communities recognize their new role as wise beings as fundamental to maintain their principles and customs. They know that safeguarding the memory is not an easy task and that it requires commitment from all members of the community. Work and oral tradition are the basic ways of teaching, learning, and transmitting knowledge and identity and that they are intimately related. The purpose of oral tradition is to instill their own knowledge in all members of the community, and this knowledge understood by the children and youngsters through their families and knowledgeable elders guarantees the identity of new generations and, subsequently, the community or people legitimized them as members of the indigenous family. From this construct, as wise indigenous “elders” and guardians of ancestral knowledge, they see their role beyond existing, and they fervently believe they have a duty to transmit, to plant for the coming generations, to create roots that will allow them to endure as a collective.

### Territoriality: Creating Roots and Uprooting

For indigenous Inga and Kamëntsá, the occupation of their territories is not only related to mercantile and economic goals, but also a way of life that integrates human being–cosmos. As mentioned previously, this indigenous communities are rooted in their territory, which goes beyond the material concept of things. Their principles are based on the thought of their cosmology and the relation of humans with the earth and earth with the spiritual. Their application of their values, the hierarchical pyramid structure that underpins their social organization, starting with spirituality and deference to their elders, determines how they interact with the outside world.

Territories allow them to develop and grow, so its absence generates instability in social and economic aspects and puts the development of their customs and experiences at risk and the loss of their identity. Due to the fact that a community’s social, economic, and cultural growth is inextricably linked to its land, a rupture with the territory results in the destruction of ancestral ways of life, social structures, languages, identities, and even the disappearance of entire communities. Identity, like the exercise of citizenship and political participation, only acquires real existence from its expression in the territory. For these communities, the loss of their territories causes uprooting, which manifests as a loss of identity, customs, discrimination, poverty, and sickness, which in turn are associated to sadness, feelings of uselessness, and a reduction of their life space: “I have always liked agriculture, but I didn’t have land to work” (CM).

## Discussion

The products of the interpretative exercise and the triangulation process and the findings’ theses are presented below.

1. The interpretation of old age for Inga and Kamëntsá communities goes beyond the number of years old; it is centered on a change of role in which they are an active part in the collective construction that has historically ruled their communities. They are active participants in the dynamics of their communities, which includes being enforcers and germinators of ancestral knowledge.

As already mention, the main premise of the symbolic interactionist view on aging is that the process of aging, as well as the changes associated with it, is socially constructed. In this vein, for these indigenous communities, aging is not associated with chronological age, although age classification systems appear to be universal, and is not seen as loss, deterioration or decline, but as a change in roles. Older people continue to be active members of society. Inga and Kamëntsá communities recognize elders as a fundamental source of traditional knowledge, and because of that, they deserve recognition. However, they also interpret their “old age” from gaining knowledge, which has happened during their life trajectory and which has awarded them a certain prestige, respect, and authority in their community. This knowledge is transmitted to reaffirm identity and preserve culture. Thus, the elder in these communities is included in their dynamics, from where they can contribute to its construction.

Indigenous knowledge is a dynamic knowledge that is recreated daily in the actions, facts, and circumstances of humans in relation to the divine, nature, family, community, and society in general. In other words, indigenous knowledge has always constituted intellectual richness to mold community individuals with identities, which is the main task assigned to wise elders (Jamioy Muchavisoy, 1997).

Having an important or main role within these communities fosters the creation of harmonic ties and the production of a social construct around the elder, who feels protected and safe. This, in turn, makes it easier to live a fulfilling life in their own terms and contributing to guiding and instructing future generations regarding customs and the culture as products of their cosmology.

All this goes against the structure of non-indigenous societies, which are mostly ruled by the economic model and measure the importance of a person in terms of production, meaning that, within their logic, when there is no production, there is no value. This was shown in the qualitative SABE Colombia study, which stated the majority of older adults feel excluded from the dynamics in the community they live in and belong to, which makes them feel uprooted. Old age in this case is being interpreted from the productive point of view; one is old when one does not work or does not produce enough (Ministerio de Salud y Protección Social - Departamento Administrativo de Ciencia Tecnología e Innovación, 2016). As opposed to this, a study of indigenous people in Ecuador revealed that their understanding of aging is shaped for this community in terms of chronic illnesses, exhaustion, deterioration of sensory capacities, vulnerability to accidents, as well as inability to work the land (Waters & Gallegos, 2014).

Further, the SABE Ecuador study (Waters & Gallego, 2012) evidenced there is a growing perception of old age as a state where opportunities, qualities, and hope are lost and that aimed at clarifying and understanding the perceptions, opinions, and concepts surrounding aging in an important part of the elder indigenous population in that country (Waters & Gallegos, 2012). The results show that for them identity is built through appreciation of the ability to perform physical activities. This aspect of identity goes against the inevitable deterioration and, as such, can affect their mental and physical health. The differences between these studies could be explained by the different methodologies, objectives and populations studied.

Blumer (1986) proposes that an individual’s sense of identity emerges from interactions with others and that interactions between various selves create societies, Therefore, from an interactionist perspective, the elderly who identifies as wise represents the norms, expectations, and values of the community that he has internalized. In the Inga and Kamëntsa communities in Putumayo, aging is strengthening their identity as knowledgeable indigenous elders: wisdom and respect are gained, their voices are heard, and they are allowed to keep their autonomy and put down more roots around what is theirs.

2. Indigenous wisdom starts with the ancestors, makes part of their cosmology, and is built through life.

For these elders, the construction of the “I am wise” starts with their involvement and personal experiences, their life trajectory, the historical memory of their people, and their cosmology. In that way, they fortify their self-perception as knowledgeable elders, and through their “oral tradition,” they try to safeguard this ancestral knowledge and their own identity.

According with Blumer(Blumer, 1986) all social behavior is “situated” and fully understandable only by reference to the context or situation in which it occurs. People create shared meanings through their interaction, and these meanings become their reality. Meaning is derived not from mental processes but from the process of interaction, and the central concern is not with how people mentally create meanings and symbols but with how they learn them during overall interaction and socialization in particular. In these communities the traditional knowledge is disseminated through oral tradition. It is grandparents and parents who teach children the ancestral heritage that has remained through the centuries. Usually, the family gathers at the end of the day and sacred tales are recounted in the Kamëntsa language.

Old age as a vital moment in human existence gathers, throughout the course of life, different experiences that are influenced by social, cultural, biological, psychological, economic, and environmental factors (Tierradentro Lasso, 2017). This materializes in the meaning of being old within the Inga and Kamëntsa communities because that process starts from the formative role of their parents and grandparents and keeps on getting stronger with those individual and collective experiences in life. In fact, these interpretations have been observed in various indigenous groups around the world and even far away from their territory, strengthened by ideas of experience, wisdom, leadership, and social value (Lagacé et al., 2012).

3. Elderly are knowledgeable-wise people who plant memory.

The knowers (Sabedores) want to signify that the knowledge of oral transmission is not produced by them, and their task is to identify it with the support of the community and then transmit it. The elderly gets their knowledge through interaction. All members of the indigenous community, including elders, women, men, youth and children, own and contribute to indigenous knowledge and this knowledge continuously transforms, evolves and adjusts as it passes from generation to generation. The Indigenous philosophy of, commonality, unity, collectivity, plurality and social justice underpins the ‘I/We’ thinking in Indigenous world, with this ‘I/We’ philosophy promoting ‘I am we’, ‘I am because we are’ and ‘a person is because of others’ relationships (Chilisa, 2012).

For the participants in the study, knowing they are wise and knowledgeable implies not only a place in the present, as discussed previously, but also a perspective of the future; their role is to strengthen the culture because they “plant memory,” contrary to the idea of finitude that is evidenced in other elder groups in Colombia (Ministerio de Salud y Protección Social - Departamento Administrativo de Ciencia Tecnología e Innovación, 2016).

The indigenous communities recognize the elders who are knowledgeable (sabedores) about traditional culture as their spokespersons and consider that their advice, opinions and recommendations are “The Correct Word”, an expression used to mean that in their words they carry deep knowledge about the subjects or objects they give to know. Thus, the indigenous knowers (sabedores) constitute a fundamental source of the traditional knowledge of their community. Inga and Kamëntsá communities consider that the life assigned by its creator continues to circulate from generation to generation, undergoing changes with the spiritual accompaniment of the elders. Because of all this, knowledgeable elders feel the need and obligation to renew their knowledge, update it and pass it on to new generations so that they do not grow up orphaned of their identity.

The indigenous knowers (sabedores) due to their extensive knowledge, have the ability to easily identify the qualities of people and the qualities of things, that is why they are generally issuing recommendations to parents so that they care for and guide their children according to the vocation or interest they have shown, as well as for the good managing things for the benefit of all. And all older people are recognized as “uncles,” creating a family atmosphere. In general, the experiences and knowledge of parents and grandparents are part of the lives of their children, which is why those transmitted from generation to generation create a certain prestige, respect and authority in the heirs.

4. Life is lived, and memory is planted in the territory.

Territory for Inga and Kamëntsá elders goes beyond physical possession, as it involves a construction from their spirituality. It is the place where they are born, they live, they learn, and where life takes meaning around their roots. For instance, for the Kamëntsa people, the territory is called Tabanok, which means “point of start and finish, to go back, to return or to come back,” and which turns into the space where knowledge about all activities they develop is exchanged: planting, seeds, handicrafts, ancestral medicine, and key foods for the survival of these communities that have been affected by many factors that place them at risk.

It is in the territory that they find what they need and desire, and territoriality overlaps the individual with the place they have lived in. There, both in their territory of origin or where the social and productive relations unfold, privileged, although not always pleasant, experiences create a referent that molds their image of self, and the aspiration facing the reality within their experience is framed.

The territory constitutes the ontological base of their identity and life. It is not possible to conceive these communities without a territory, because it is the cultural container and the experiential framework for spiritual, social, cultural, political, and economic recreation and for strengthening their cosmology. There are intimate relationship and connectedness of Indigenous peoples experience with nature and with the territory as a result of their worldviews. Thus, it should not be expressed solely in material terms (Báez-Manrique, 2017).

5. The Inga and Kamëntsa indigenous communities from Valle de Sibundoy in Putumayo may come from different roots, but they are linked by means of the land that shelters them and are a living example of respect for others and nature. During the building of their communities that sometimes intertwine, they share some qualities: elders are heard and valued.

The Inga and Kamëntsa indigenous communities have historically coexisted in the same territory; as was described in previous pages, they come from different roots, have different languages, and have their own customs. Nevertheless, despite these differences, they have coexisted peacefully and in harmony for many years, which has, in turn, strengthened both communities. As such, they share some qualities, among which we can find how they value and take into account the role of the elders (Pinzón & Garay, 1996).

This study has a number of strengths. Qualitative research methods were used because they help to comprehend perceptions, opinions, and concepts that influence individual and collective behavior. We argue that the SI share aspects with the Indigenous Inga and Kamëntsa cosmology. Specifically, and importantly, this framework highlights how reality is constructed through interactions between and within peoples. Consequently, the combining of the indigenous cosmology and SI suggest can enable researchers to respect and appreciate diverse Indigenous interactions, and cultural diversity, facilitate other ways of knowing, and could promote indigenous participation in research. This micro perspective of indigenous aging is a unique lens through which we can observe diverse aging processes. It also requires us to focus considerably more on providing culturally relevant that aids in the development of differentiated public policies. This study also has some limitations. Given the character of this study and the small convenience sample used, conclusions cannot be generalized to the full Inga and Kamëntsa communities. Another limitation is that the interviews were conducted in Spanish, which was not the participants’ first language. The interpretation and linguistic forms of the interviewer and participants can alter symbolic communication.

## Conclusions

The purpose of this study was to elucidate the meaning of aging for two indigenous communities in southern Colombia, thereby generating a new perspective that differs from the concept of aging and its implications in non-indigenous communities and emphasizing the critical importance of the aging process in these communities. Whereas the elderly are regarded as the bearers of invaluable ancestral knowledge passed down through generations, they are also charged with the responsibility of preserving and transmitting this knowledge in order to safeguard their cultural identity in close interaction with the territory, and the associated factors that lead to uprooting, these findings highlight the importance of recognizing and valuing their primary role within these communities.

From this study, we can conclude that interpreting old age for these communities does not follow the logic of age or getting older, or physical capacity, or health, sickness (Moody & Sasser, 2020), but it centers on a new role (a change of role) that makes it possible to visualize themselves as wise beings. Moreover, wisdom is built in parallel with their cosmology and assigns them the task of safekeeping ancestral knowledge. In order to do this, they use oral tradition as a tool, words that are born in their territories, travel in a nonlinear timeline, and get strengthened by the community while also protecting it and building it.

Aging and old age are universal realities, however, All the ideas and notions that we associate with aging can be diverse and divergent, have been created by members of society. Aging, among indigenous Inga and Kamëntsa, has a connotation of wisdom and carries social respect in contrast to non-indigenous perspectives. This notions, as socially constructed realities in these two indigenous peoples, are not generalizable or applicable to other groups or other times or spaces.

From the interpretation of old age for these communities, becoming wise invites them to keep their cosmology, customs, and territory alive and to strengthen their identity. All this creates roots, and on the contrary, the loss of this wisdom and the elements that make part of it uproot them and leave them at risk of disappearing as individuals and as a people.

The results of this study make it possible to understand the peculiarities of aging and old age in these indigenous communities, which differ from what other groups report. Thus, all this knowledge should be taken into account when creating projects and policies that want to have an impact on the communities, but want to preserve what has been so hard for them to maintain: their cosmology.

This research is pioneer in the country and shows another perspective of old age. Knowing what aging means for Indigenous communities can facilitate to the development of policies and initiatives and to provide culturally appropriate and effective programs.

## Data Availability

The datasets generated by the survey research during and/or analyzed during the current study are available in this repository: https://www.kaggle.com/datasets/hernangarciabotina/analysis-of-data?utm_medium=social&utm_campaign=kaggle-dataset-share&utm_source=facebook&fbclid=IwAR2E2U7yy_FQlHHzpFyPtX9DkYijVHXVT4ySlueF5pXVndEUbHh7-CZ6_l8.

## Footnotes

1 Textual accounts of the participants are identified with letters that indicate their activity in the community: P (politician), TD (traditional doctor), A (artisan), M (musician), CW (community woman), CM (community man).
